# Passaging Human Tauopathy Patient Samples in Cells Generates Heterogeneous Fibrils with a Subpopulation Adopting Disease Folds

**DOI:** 10.1101/2023.07.19.549721

**Published:** 2025-01-02

**Authors:** Zhikai Zeng, Karen Tsay, Vishnu Vijayan, Matthew P. Frost, Shikhar Prakash, Athena Quddus, Alexa Albert, Michael Vigers, Madhur Srivastava, Amanda L. Woerman, Songi Han

## Abstract

The discovery by cryo-electron microscopy (cryo-EM) that the neu-ropathological hallmarks of different tauopathies, including Alzheimer’s disease, corticobasal degeneration (CBD), and progressive supranuclear palsy (PSP), are caused by unique misfolded conformations of the protein tau is among the most profound developments in neurodegenerative disease research. To capitalize on these discoveries for therapeutic development, one must achieve *in vitro* replication of tau fibrils that adopt the representative tauopathy disease folds, which represents a grand challenge for the field. A widely used approach has been seeded propagation using pathological tau fibrils derived from post-mortem patient samples in biosensor cells that expresses a fragment of the tau protein known as K18, or Tau4RD, containing the microtubule-binding repeat domain of tau as the substrate. The new insights from cryo-EM raised the question of whether the Tau4RD fragment is capable of adopting characteristic tau folds found in CBD and PSP patient fibrils, and whether cell-passaged and amplified tau fibrils can be used as seeds to achieve cell-free assembly of recombinant 4R tau into fibrils without the addition of cofactors. Using Double Electron Electron Resonance (DEER) spectroscopy, we discovered that cell-passaged pathological seeds generate heterogeneous fibrils that are, however, distinct between the CBD and PSP lysate-seeded fibrils, and vastly different from heparin-induced tau fibril structures. Moreover, the lysate-seeded fibrils contain a characteristic sub-population that resembles the disease fold corresponding to the respective starting patient sample. These findings indicate that templated propagation using CBD and PSP patient-derived fibrils is possible with a tau fragment that does not contain the entire pathological fibril core, but also that additional mechanisms must be tuned to converge on a homogeneous fibril population.

## Full Text Availability

The license terms selected by the author(s) for this preprint version do not permit archiving in PMC. The full text is available from the preprint server.

